# Impaired Coupling between the Dorsomedial Prefrontal Cortex and the Amygdala in Schizophrenia Smokers Viewing Anti-smoking Images

**DOI:** 10.3389/fpsyt.2017.00109

**Published:** 2017-06-19

**Authors:** Stéphane Potvin, Andràs Tikàsz, Ovidiu Lungu, Emmanuel Stip, Vesséla Zaharieva, Pierre Lalonde, Olivier Lipp, Adrianna Mendrek

**Affiliations:** ^1^Department of Psychiatry, University of Montreal, Montreal, QC, Canada; ^2^Centre de recherche de l’Institut Universitaire en Santé Mentale de Montréal, Montreal, QC, Canada; ^3^Centre de Recherche de l’Institut Universitaire de Gériatrie de Montreal, Montreal, QC, Canada; ^4^Centre for Research in Aging, Donald Berman Maimonides Geriatric Centre, Montreal, QC, Canada; ^5^Montreal Neurological Institute and Hospital, McGill University, Montreal, QC, Canada; ^6^Department of Psychology, Bishop’s University, Sherbrooke, QC, Canada

**Keywords:** schizophrenia, smoking, harms, functional magnetic resonance imaging, connectivity

## Abstract

**Background:**

Cigarette smoking is highly prevalent in schizophrenia and is one of the main factors contributing to the significantly decreased life expectancy in this population. Schizophrenia smokers, compared to their counterparts with no comorbid psychiatric disorder, are largely unaware and indifferent to the long-term negative consequences of cigarette smoking. The objective of this study was to determine, for the first time, if these meta-cognitive deficits are associated with neuro-functional alterations in schizophrenia smokers.

**Methods:**

Twenty-four smokers with no psychiatric disorder and 21 smokers with schizophrenia (DSM-IV criteria) were scanned using functional magnetic resonance imaging and exposed to anti-smoking images. Granger causality analyses were used to examine the effective connectivity between brain regions found to be significantly activated.

**Results:**

Across groups, potent activations were observed in the left ventro-lateral prefrontal cortex, the left amygdala (AMG), and the dorsomedial prefrontal cortex (dmPFC). Using the dmPFC as a seed region, we found an abnormal negative connectivity from the dmPFC to the AMG in schizophrenia smokers during the viewing of anti-smoking stimuli. This abnormal connectivity was not present during the viewing of aversive stimuli unrelated to tobacco.

**Discussion:**

Given the well-established roles of the dmPFC in social cognition and of the AMG in emotional processing, our results suggest that the relative indifference of schizophrenia smokers regarding the negative consequences of tobacco smoking could be explained by a cognitive-affective dissonance.

## Introduction

As shown in a meta-analysis of worldwide studies, there is a threefold to sixfold increase of the prevalence (current and lifetime) of cigarette smoking in schizophrenia (1). In these patients, tobacco smoking has dramatic effects on their health. For instance, the risk of cardiac-related death has been shown to be increased by 12-fold in smokers with schizophrenia, relative to schizophrenia patients who do not smoke (2). Unfortunately, schizophrenia smokers achieve lower cessation rates than smokers with no comorbid psychiatric disorders (3). Despite the fact that tobacco smoking is highly prevalent and has harmful effects in schizophrenia, the mechanisms involved in the patients’ motivation to smoke are inadequately understood.

The lead explanation for the increased prevalence of tobacco smoking in schizophrenia is the self-medication hypothesis, which postulates that these patients smoke cigarettes in order to relieve their symptoms, their cognitive deficits, and/or the side effects of their medication (4–6). Although some studies have shown that nicotine improves cognitive functioning in schizophrenia (7), others have failed to replicate these findings (8, 9). Moreover, some authors have criticized the self-medication hypothesis for its implicit justification of tobacco smoking in schizophrenia (10). While several studies have been undertaken to prove or disprove the self-medication hypothesis, it is striking to observe that considerably less attention has been paid to the fact that schizophrenia patients seem largely unaware of the harmful effects of cigarette smoking. In a rare study on the topic, Kelly et al. (11) assessed the perceived harmful consequences of tobacco smoking in 100 schizophrenia-spectrum smokers and 100 control smokers, using the *Smoking Consequences Questionnaire*, and found that psychotic smokers underestimated the health risks associated with smoking. Likewise, in a study involving 1,046 participants who were exposed to anti-substance use campaigns, Thornton et al. (12) found a negative association (trend level) between psychosis and risk perceptions for tobacco. Such preliminary results may be explained by the memory deficits, the emotional flattening, and/or the poor meta-cognitive abilities (e.g., insight) associated with schizophrenia (13–15) and may potentially explain the low quitting rates observed in these patients (3).

In recent years, a growing number of functional magnetic resonance imaging (fMRI) studies have examined how cigarette smokers (with no comorbid psychiatric disorder) respond to smoking-cessation messages or to stimuli displaying the harmful effects of tobacco. Thus far, it has been consistently shown that anti-smoking stimuli elicit activations in the medial prefrontal cortex and the amygdala (AMG) in chronic smokers (16–20). Whereas the AMG activations are likely to underlie the emotional response produced by the anti-smoking stimuli (19), the medial prefrontal activations seems to play a role in the processing of the self-relevancy of these aversive stimuli (16, 21). Importantly, activations in both regions in response to anti-smoking stimuli have been shown to predict changes in smokers’ future behavior, as measured by successful quitting rates or urine cotinine levels (16, 18–20). Such neuroimaging results echo the results of several intervention studies having shown that the most efficient anti-smoking messages do not only elicit an emotional response in smokers but also make them feel *personally* concerned (22, 23).

Despite these promising and relevant findings, we are unaware of any functional neuroimaging study having examined how schizophrenia smokers respond to anti-smoking images, even though these patients are more likely to smoke, have more difficulties quitting, and are most probably less aware of the health risks associated with tobacco smoking. In the past, several fMRI studies have shown that schizophrenia patients have abnormal fronto-limbic activations in response to aversive stimuli unrelated to tobacco (24). Moreover, a growing body of work shows that the connectivity between the prefrontal cortex and limbic regions is impaired in schizophrenia during the processing of negative emotional stimuli (25, 26). An important knowledge gap that remains to be address is whether schizophrenia patients also have altered fronto-limbic connectivity in response to stimuli elicited negative emotional experiences that are *specifically* related to the harmful consequences of tobacco smoking. This study sought to test this general hypothesis and address this key knowledge gap. For that purpose, we used fMRI to compare how schizophrenia smokers respond to anti-smoking images, relative to smokers with no comorbid psychiatric disorder, using (lagged) connectivity analyses.

## Materials and Methods

### Participants

Twenty-one smokers with schizophrenia (*n* = 18) or schizo-affective disorder (SCZ) (*n* = 3), and 23 smokers with no co-occurring psychiatric disorder (DSM-IV) were recruited. In the same study, we also investigated craving. For results, please refer to Ref. (27). In both groups, participants were smoking ≥10 cigarettes per day. None of the smokers were currently receiving pharmacological aid for smoking cessation. Smokers with SCZ had no co-occurring substance use disorder (other than tobacco) in the last 12 months, as determined by psychiatric interview and confirmed by negative urine drug screenings. SCZ smokers were recruited at the *Institut Universitaire en Santé Mentale* (Montreal, QC, Canada). Other than tobacco use disorder, control smokers had no other axis I or II psychiatric disorder (including substance use disorder), and none were taking psychiatric or neurologic medications. The recruitment of control smokers was done through the hospital and its affiliated research center as well as advertisements on the Internet. In both groups, smokers had no neurologic disorders, no unstable medical problem, and no contra-indications for MRI. Further description of the sample can be found in Table 1. SCZ smokers were outpatients stabilized on antipsychotic medication that had not changed within the last 2 months. They were treated with one or more of the following antipsychotics: aripiprazole (*n* = 3); clozapine (*n* = 12); olanzapine (*n* = 4); quetiapine (*n* = 2); risperidone (*n* = 3); fluphenazine (*n* = 1); zuclopenthixol (*n* = 2); perphenazine (*n* = 1); ziprasidone (*n* = 1).

**Table 1:** Demographical, clinical, and behavioral data.

Variable	Healthy controls (*n* = 23)	Schizophrenia patients (*n* = 21)	Statistics
**Sociodemographic data**			
Age	33.2 (10.1)	34.8 (8.8)	*F* = 0.31; *p* = 0.58
Sex (males)	15	16	χ^2^ = 0.64; *p* = 0.43
Education (years)	12.7 (2.57)	11.3 (2.0)	*F* = 3.79; *p* = 0.06
Handedness (right)	22	19	χ^2^ = 0.46; *p* = 0.50
**Smoking habits**			
Age of onset	16.3 (3.7)	15.9 (4.9)	*F* = 0.10; *p* = 0.76
Number of cigarette/day	20.3 (5.6)	19.0 (5.2)	*F* = 0.64; *p* = 0.43
Number of attempts to quit	3.0 (2.9)	3.0 (3.0)	*F* = 0.00; *p* = 0.96
FTND	5.0 (2.4)	6.0 (1.8)	*F* = 2.67; *p* = 0.11
FTCQ-12	3.8 (1.1)	4.1 (0.8)	*F* = 0.71; *p* = 0.41
**Emotional intensity ratings**			
Neutral	10.3 (12.9)	21.1 (18.5)	***F* = 5.08; *p* = 0.03**
Negative	68.5 (19.6)	66.5 (21.9)	*F* = 0.11; *p* = 0.75
Tobacco	60.5 (22.3)	58.5 (23.2)	*F* = 0.09; *p* = 0.77
**Clinical data**			
BDI-II	5.3 (5.9)	11.4 (8.5)	***F* = 7.30; *p* = 0.01**
**PANSS**			
Positive	−	16.0 (3.7)	−
Negative	−	15.7 (4.5)	−
General	−	36.4 (5.7)	−
**Chlorpromazine equivalents**			
Mg	−	601 (360)	−

*BDI, Beck Depression Inventory; PANSS, Positive And Negative Syndrome Scale; FTND, Fagerström Test for Nicotine Dependence; FTCQ-12, French Tobacco craving questionnaire; Numbers in parentheses represent SD*.

*Bold font indicates significant between-group differences*.

This study was carried out in accordance with the recommendations of the *Regroupement Neuroimagerie Québec* with written informed consent from all subjects. All subjects gave written consent in accordance with the Declaration of Helsinki. The protocol was approved by the *Regroupement Neuroimagerie Québec*.

### Clinical Assessments

In both groups of smokers (with and without SCZ), the *Fagerström Test for Nicotine Dependence* (28), the *French Tobacco Craving Questionnaire* (29), and the *Beck Depression Inventory*-II (BDI) (30) were used to assess tobacco use disorder severity, cigarette cravings, and depressive symptoms, respectively. In smokers with SCZ, psychiatric symptoms (positive and negative) were evaluated with the *Positive and Negative Syndrome Scale* (31).

### fMRI Procedures

In order to standardize the experimental procedure, participants were invited to smoke one last cigarette 30–40 min prior to the fMRI scanning session. To make sure that smokers paid attention to smoking-related stimuli while they were in the scanner, they were asked to press a button each time a new picture appeared. Participants viewed an alternating sequence of aversive (smoking and non-smoking-related) and neutral images. The aversive smoking-related images consisted of unpleasant and arousing images illustrating the negative consequences of smoking, such as ill health, death, and addiction (e.g., lung cancer, skull smoking, and a person trapped in a cigarette). Every picture contained a cigarette and no text was included. The aversive non-smoking-related and neutral pictures were selected from the *International Affective Picture System* (IAPS) (32). Aversive images (tobacco-related or not) were matched in valence and arousal based on a pilot study performed in 50 individuals. Images were also matched for the number of colors, visual complexity, the number of faces, and the proportion of body parts. For a detailed description of the stimuli previously presented in control smokers, see Dinh-Williams et al. (17).

**Figure 1:**
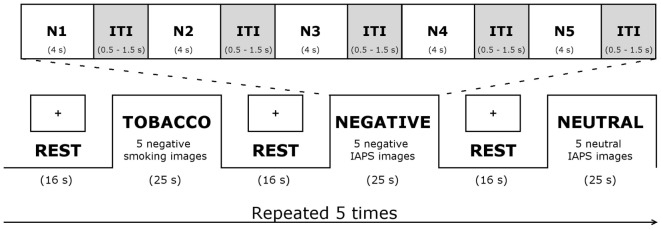
Functional magnetic resonance imaging task. Abbreviations: IAPS, International Affective Picture System; ITI, inter-stimulus interval; N1–5, 5 negative pictures.

The task comprised an alternating sequence of five aversive smoking-related, five aversive (IAPS) non-smoking-related, and five neutral blocks (see Figure 1). Blocks were separated from one another by rest periods, consisting of a fixation cross displayed on a blank screen for 16 s. Each experimental block lasted 25 s, during which five pictures were presented, for 4 s each, with a mean inter-stimulus interval of 1 s. Across blocks, smokers viewed a total of 75 pictures (25 pictures for each of the 3 experimental conditions). At the end of the scanning session, smokers were presented once again with the 75 images. They had to rate the pictures on a visual analog scale from 0 (no smoking desire) to 100 (strongest smoking desire).

### Neuroimaging Acquisition Parameters

Whole-brain fMRI was performed using an echo-planar imaging (EPI) sequence measuring blood oxygenation level dependent (BOLD) signal (TR = 3,000 ms; TE = 30 ms; FA = 90°; matrix size = 64 × 64; voxel size = 3.5 mm^3^; 41 slices) on a Siemens Trio Tim MRI 3-T system. The 210 functional slices were oriented in transverse plane and were angled to be parallel to the AC–PC line. An inline retrospective motion correction algorithm was employed, while the EPI images were acquired. Individual high-resolution co-planar anatomical images were also acquired during the same scanning session (three-dimensional, ultrafast gradient echo sequence; TR = 2,300 ms, TE = 2.98 ms, flip angle = 9°, matrix size = 256 × 256, number of slices = 176, voxel size = 1.0 mm^3^).

### fMRI Data Analysis

Functional magnetic resonance imaging data were preprocessed and analyzed using BrainVoyager QX software (Brain Innovation, Maastricht, Netherlands). Functional images were corrected for the difference in slice-time acquisition, corrected for motion artifacts (movements in any direction ≤ 2 mm), high-pass filtered (two cycles per time course) to correct for signal drift, co-registered to the corresponding T1 volume, normalized to the stereotaxic Talairach space (33), and spatially smoothed using an 8-mm Gaussian kernel.

#### fMRI Analyses of Brain Activations

Data analysis was performed using a blocked-design approach. We defined three predictors, corresponding to the blocks of aversive smoking-related (Tobacco), aversive non-smoking-related (Negative), and neutral images (Neutral). At the individual level, the three predictors were entered as fixed factors in a general linear model (GLM). Then, group analyses were performed by entering the parameters of first-level GLM into a second-level analysis corresponding to a random-effect model (34). An autoregressive AR(1) model was used to account for serial correlations. The GLM was estimated for each voxel brain-wise and was used to detect brain regions, where the activation (BOLD signal) was stronger during Tobacco than Neutral condition across (schizophrenia patients and controls, combined) and between groups. For the analyses across groups, significantly activated clusters were displayed using a minimum size of 900 contiguous voxels (900 mm^3^; surpassing 20 non-resampled voxels), and a stringent statistical threshold of *t*(43) = 4.0 [surpassing *q*(False Discovery Rate) < 0.005]. We identified brain regions activated across groups instead of using brain regions found to differ between groups, as the latter approach is associated with an implicit bias that increases the odds of finding group differences in connectivity analyses (35). Of all the regions found to be significantly activated for the (Tobacco > Neutral) contrast, we selected the prefrontal and limbic regions for the lagged connectivity analyses, as they are key regions to self-processing, emotion regulation, and aversive response.

#### Granger Causality (GC) Analysis

We employed Granger causality modeling (GCM) to determine if immediate past values from the time series of one brain region predict the current values from the time series of another region, while controlling for the confounding effect from its own immediate past values. The GCM method was developed in the economics field (36) in order to account for temporal dependencies in time series. Since then, GCM has been successfully used in investigating the lagged connectivity between neural time series of multiple brain regions (37–39), and it has become increasingly used in the field (37).

In this study, we implemented GCM by first extracting the raw (BOLD) time course from the peak of the regions of interest (RIOs) resulting from the contrast (Tobacco > Neutral). Then, these time series were converted into *z*-scores (mean = 0, SD = 1). In order to account for modulation in Granger effective connectivity by the task, we used an approach similar to the psychophysiological interaction (PPI) technique (40), whereby we included in the regression model the values of the BOLD signal time series originating from a seed cluster multiplied with the weights of the regressor corresponding to the task (i.e., the PPI term). This approach allowed to evaluate the variations in GC effective connectivity between pairs of ROIs during experimental conditions (i.e., Tobacco, Negative, and Neutral) relative to one another or to rest periods (26, 41).

Considering A and B, a pair of ROIs, the GCM from A to B is expressed by the following univariate multiple regression equation:

$$Y_{t}=\beta_{0}+\beta_{1}Y_{t-1}+\beta_{2}X_{t-1}+\beta_{3}X_{t-1}*\left(\text{Tobacco}\right)+\beta_{4}X_{t-1}\text{*}\left(\text{Other}\right)+\varepsilon$$

where, *Y_t_* = BOLD signal in the target region (B) at time *t*; *Y_t−_*_1_ = BOLD signal in the target region (B) at time *t* − 1 (autoregressive component); *X_t−_*_1_ = BOLD signal in the seed region (A) at time *t* − 1 (GCM predictor); *X_t−_*_1_*(Tobacco) = the interaction between the BOLD signal in the seed region (A) and a vector corresponding to the Tobacco experimental condition (GCM-PPI predictor); *X_t−_*_1_*(Other) = the interaction between the BOLD signal in the seed region (A) and a vector corresponding to the other remaining experimental conditions (GCM-PPI predictor).

The coefficient of interest in this equation is β_3_*X_t−_*_1_*(Tobacco). It is a measure of the effective lagged connectivity from A (at *t* − 1) to B (at *t*) during the Tobacco condition. Once the GCM are estimated in both directions (from A to B and from B to A), the directionality of the Granger-causal connectivity is determined by subtracting the β_3_ of one model (A to B) from the corresponding β_3_ of the other model (B to A), for the same participant (i.e., difference GCM index or dGCM). In our GCM analyses, we estimated the GC coefficients for each stimulus type (separately for Tobacco, Negative, and Neutral), and concentrated on between-group differences in dGCM within each stimulus type. Between-group differences in GC coefficients from the dGCM (A to B minus B to A) were examined using random-effect *F*-tests. In the case of significant differences, we further examined the GC coefficients for each GCM separately (A to B versus B to A). One-sample tests were used to determine whether the GC coefficients were different from 0 at the group level.

Finally, potential differences between groups in clinical variables were assessed using chi-square tests for discrete data, and analyses of variances for continuous data. We also performed correlation analyses to examine the potential associations between GC coefficients, participants’ emotional ratings, and patients’ psychiatric symptoms and antipsychotic dosage. For these analyses, we only used GC coefficients for which between-group differences were observed. The statistical threshold for rejecting the null hypothesis was set at *p* < 0.05, and we applied a Bonferroni correction.

## Results

### Demographic, Clinical, and Behavioral Data

As illustrated in Table 1, schizophrenia patients and healthy controls did not differ in terms of age, sex, smoking habits (age of onset, number of cigarettes per day, number of quitting attempts, baseline cravings, and nicotine dependence severity), and ratings of smoking non-related (Negative) and smoking-related (Tobacco) images. However, schizophrenia patients rated neutral images as more emotional and reported more depressive symptoms on the BDI scale than healthy controls did.

### Brain Regions Specific to Aversive Smoking-Related Images

**Figure 2:**
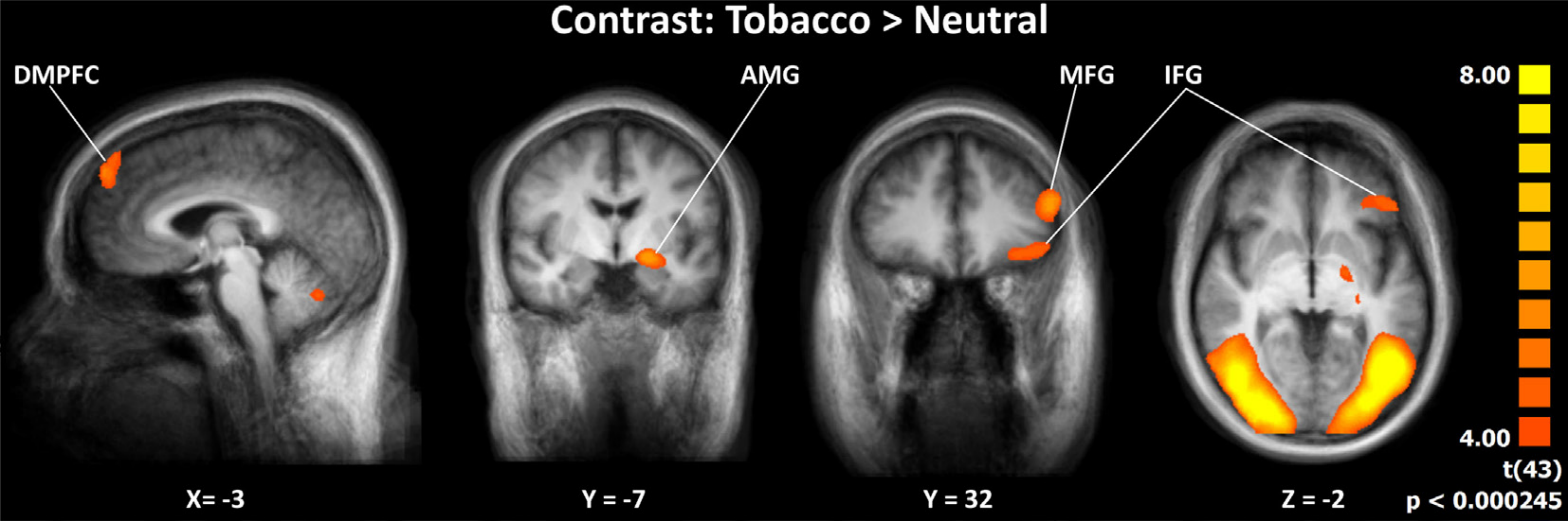
Activation specific to aversive smoking-related images across groups. Abbreviations: DMPFC, dorsomedial prefrontal cortex; AMG, amygdala; MFG, middle frontal gyrus; IFG, inferior frontal gyrus. Images displayed in radiological convention.

Seven brain regions were significantly activated by the (Tobacco > Neutral) contrast across the two groups: the left dorsomedial prefrontal cortex (dmPFC), the left AMG, the left inferior frontal gyrus (IFG), the left middle frontal gyrus (MFG), the bilateral occipital gyrus/fusiform gyrus, and the cerebellum (Figure 2). Of these regions, the dmPFC, AMG, IFG, and MFG were selected to conduct further GCM analyses, as they have been shown to be involved in the processing of anti-smoking images in previous studies (16, 18–20). For coordinates, clusters sizes, and BOLD signal, see Table S1 and Figure S1 in Supplementary Material.

### Granger-Causal Connectivity: Group Differences within Each Condition

**Figure 3:**
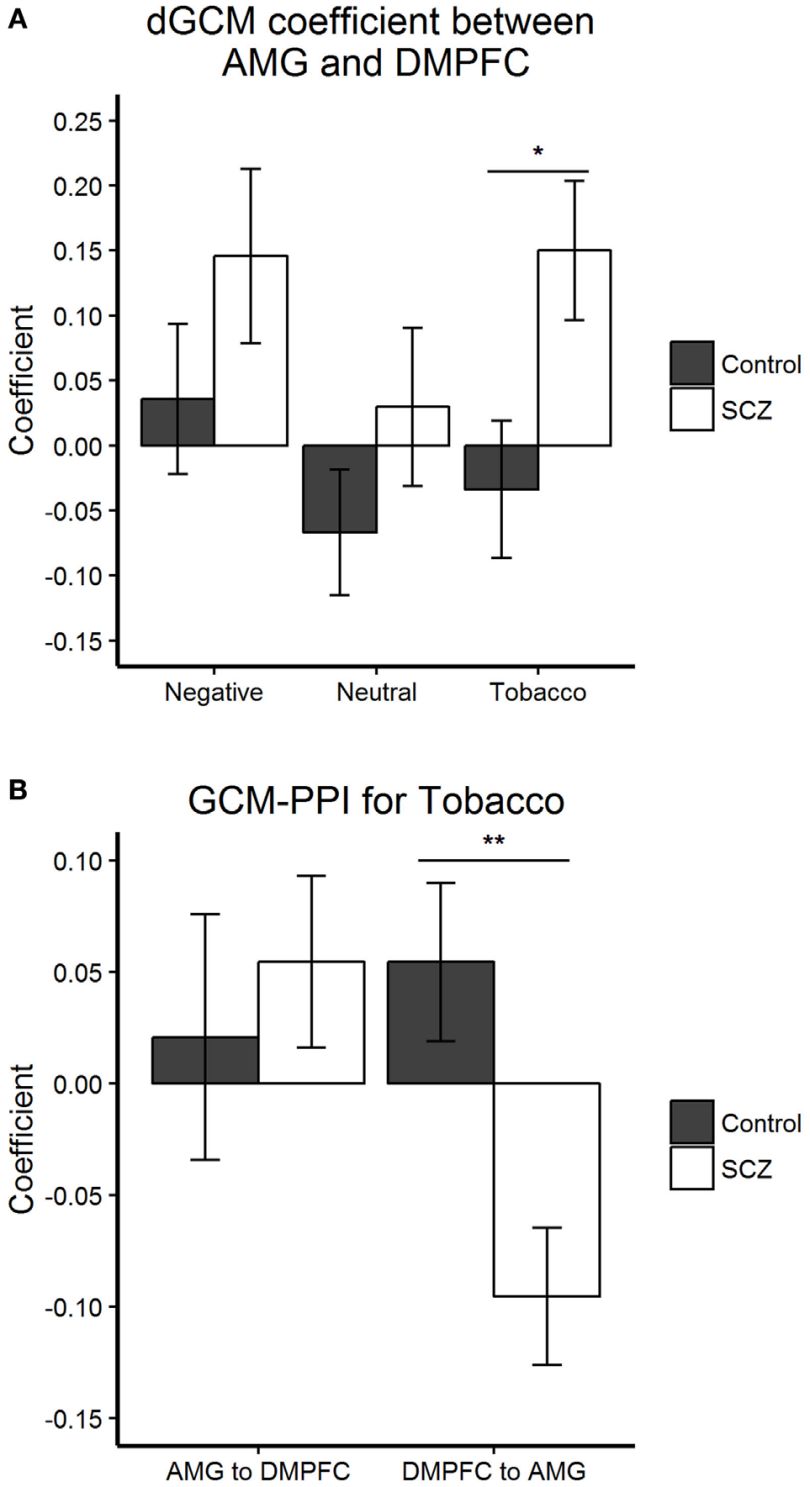
Granger-causal connectivity: group differences within each condition. **(A,B)** Bar plots display the mean and SEM. **(A)** Condition-by-condition analysis revealed a significant dGCM (A to B minus B to A) (*F*_1,42_ = 5.962, *p = 0.019) group difference during aversive smoking-related stimuli (Tobacco) for the connectivity between left AMG and dmPFC.**(B)** The autoregressive Granger causality modeling (GCM)-psychophysiological interaction (PPI) components underlying dGCM allowed to investigate the lagged connectivity from the AMG to DMPFC and from DMPFC to AMG separately, during the Tobacco condition. The latter revealed a significant (*F*_1,42_ = 10.039, **p = 0.003) between-group difference in lagged connectivity from the DMPFC to the AMG during viewing of aversive smoking-related stimuli (Tobacco). Abbreviations: AMG, left amygdala; DMPFC, dorsomedial prefrontal cortex; SCZ, schizophrenia patients; Control = healthy control participants.

Granger-causal connectivity was tested between three pairs of regions: dmPFC-AMG, IFG-AMG, and MFG-AMG. Condition-by-condition analysis revealed a significant dGCM (region A to region B minus region B to region A) between-group difference during aversive smoking-related stimuli (Tobacco) for the connectivity between left AMG and dmPFC (Figure 3A). The autoregressive GCM-PPI components underlying dGCM allowed to investigate the lagged connectivity from the AMG to dmPFC and from dmPFC to AMG separately, and for each stimuli condition (Figure 3B). The latter revealed a significant between-group difference in lagged connectivity from the dmPFC to the AMG during viewing of aversive smoking-related stimuli. Further analysis showed a significant negative lagged connectivity from the dmPFC to the AMG in schizophrenia patients [*t*(20) = −3.109, *p* = 0.006], and a non-significative positive lagged connectivity from the dmPFC to the AMG in healthy controls [*t*(22) = 1.536, *p* = 0.139]. There were no between-group differences in dmPFC-AMG connectivity for the Negative and the Neutral conditions (see Figure 3).

Finally, the analyses on the lagged connectivity between the IFG and the AMG, as well as between the MFG and the AMG, did not reveal between-group differences that surpassed the statistical threshold (*p* > 0.05).

### Granger-Causal Connectivity: Correlations with Clinical Variables

We found no significant relationships between GC coefficients or dGCMs and participants’ smoking habits (age of onset, number of cigarettes per day, number of quitting attempts, baseline cravings, and nicotine dependence severity), schizophrenia patients’ psychiatric symptoms (positive, negative, and depressive symptoms), and patient’s antipsychotic dosage (chlorpromazine equivalents). GC coefficients and dGCM did not differ between schizophrenia patients on and off clozapine. We did observe that the ratings of aversive smoking-related images (Tobacco) correlated with the dGCMs for the Tobacco stimuli in healthy controls (*r* = 0.431, *p* = 0.040) and not in schizophrenia patients (*r* = −0.252, *p* = 0.271). However, this correlation did not survive a Bonferroni correction. Noteworthy, the ratings of neutral images did not correlate with the dGCMs in both groups of smokers.

## Discussion

In view of the low quitting smoking rates in schizophrenia and the relative unawareness of these patients of the harmful consequences of cigarette smoking, this study sought to examine the neural response to anti-smoking images of smokers with schizophrenia, compared to those with no co-occurring psychiatric disorder. Across groups, we found that the images displaying the health risks of tobacco smoking elicited potent activations of the dmPFC, the left ventro-lateral prefrontal cortex, the left AMG, and other bilateral temporo-occipital regions. More importantly, we found that the connectivity between the dmPFC and the left AMG was impaired in schizophrenia smokers. There were no differences in emotional ratings of anti-smoking stimuli, but schizophrenia patients rated neutral stimuli as being more emotional than control smokers did. In control smokers only, there was a moderate positive correlation between emotional ratings of the anti-smoking images and the AMG-dmPFC connectivity. However, this correlation did not survive Bonferroni correction. Finally, schizophrenia smokers had increased depressive symptoms, compared to control smokers, but depressive symptoms had no influence on connectivity results.

Anti-smoking images elicited in both groups of smokers potent activations in the dorsomedial prefrontal cortex and the left AMG. As such, these results are clearly consistent with the results of the first fMRI studies performed on the neural mechanisms involved in the processing of the anti-smoking messages. These results suggest that the anti-smoking images recruit regions that are thought to play a key role in the emotional responding to these images (e.g., the AMG) (19), as well as the processing of their self-relevancy (e.g., the dorso-/medial prefrontal cortex) (16, 21). Classical fMRI analyses also revealed significant activations across groups of smokers in the ventro-lateral and the lateral prefrontal cortex in response to aversive tobacco-related stimuli. In the past, several fMRI studies have shown that the ventro-/lateral prefrontal cortex are significantly activated when individuals are viewing aversive images (such as emotional faces) unrelated to the harmful consequences of tobacco smoking (42). Further fMRI studies on emotional regulation have revealed, more precisely, that the ventro-/lateral prefrontal cortex are involved in the executive control exerted over the negative emotional experience (43, 44). Taken together, these results suggest that the activations of the ventro-/lateral prefrontal cortex observed in both groups of smokers reflect an attempt to cope with the negative emotions triggered by the viewing of the anti-smoking images.

The novel and the most important result of this study is the finding of an impaired fronto-limbic connectivity in schizophrenia smokers, relative to control smokers. When examining the main GC model (AMG to dmPFC minus dmPFC to AMG), we found an increased connectivity in schizophrenia smokers, compared to control smokers. Further sub-analyses showed that this effect was actually explained by the presence of a negative connectivity from the dmPFC to the AMG in schizophrenia smokers, an effect that was not present in control smokers. Apart from its well-established role in the processes involved in self-other distinction (45), the dmPFC also seems to play a role in action selection (46, 47). It is also possibly involved in emotion regulation, although results in this case are less consistent (43, 44, 48). As such, this suggests that the dmPFC, a region involved in elaborate cognitive processes, has an aberrant inhibitory influence, in schizophrenia smokers, on the response of the AMG to images displaying the harmful health effects of tobacco smoking. Regardless of the exact meaning of this aberrant negative connectivity from the dmPFC to the AMG in schizophrenia smokers, we can safely assume that it highlights a cognitive-affective dissonance which may underlie the indifference of schizophrenia patients toward the negative value of tobacco smoking. Consistently with the idea of a cognitive-affective dissonance, the AMG-dmPFC connectivity (main GCM) was positively correlated with emotional ratings of anti-smoking images in control smokers, but this was not the case in schizophrenia smokers.

In the same sample of participants, we previously examined how they responded to appetitive smoking images designed to elicit cigarette cravings. Interestingly, we found that schizophrenia smokers had increased activations in the (bilateral) ventro-medial prefrontal cortex (vmPFC), relative to control smokers, when viewing appetitive smoking images (27). Given that the vmPFC is a key region of the brain reward system (49), the results of the previous showed that schizophrenia is characterized by a state of sensitization to the rewarding effects of tobacco. Taken together with the results of this report, the results of both investigations suggest the motivational value of cigarette smoking is imbalanced in the schizophrenia brain. That is, whereas the brain reward system of schizophrenia patients seems to be sensitized to the *appetitive* value of smoking, the neural pathway involved in the processing of the *aversive* value of smoking appears disconnected. In theory, both mechanisms could explain why schizophrenia smokers achieve lower quitting rates.

The most important limitation of this study is that a fair proportion of schizophrenia smokers were treated with clozapine (*n* = 12), meaning that our sample of patients was composed of a significant proportion of treatment-resistant patients with schizophrenia. As such, the interpretation of our results should be taken prudently, given that our sample of participants may not be representative of the schizophrenia population. However, a recent systematic review showed that there is an association between treatment resistance and tobacco smoking (50). Moreover, we performed sub-analyses and found that patients on and off clozapine did not differ in the lagged connectivity between the AMG and the dmPFC. Another limitation of our study is that smokers with and without schizophrenia were not asked if the tobacco-related aversive images made them crave for cigarettes. Finally, even if the validity of GC analyses is increasingly acknowledged in the neuroimaging field (51), the interpretation of the results from these analyses can be partially hindered by differences in the latency in hemodynamic response function between brain regions (39). Conversely, one of the strengths of this study is that we included an experimental condition composed of images designed to elicit aversive emotional responses unrelated to tobacco smoking. Importantly, we found no altered connectivity in schizophrenia smokers, relative to control smokers, in the Negative and Neutral condition, suggesting that the impaired lagged connectivity from the dmPFC to the AMG is *specifically* impaired when patients are processing anti-smoking images. However, we cannot rule out that the recruitment of a larger sample of participants may have revealed altered connectivity during the Negative condition as well.

This is probably the first fMRI study to investigate the neural mechanisms involved in the relative indifference of schizophrenia smokers regarding the health risks associated with tobacco smoking (at least, to our knowledge). Results revealed the presence of a negative lagged connectivity from the dmPFC to the AMG in schizophrenia smokers who viewed anti-smoking images in the scanner. These results suggest that a cognitive-affective dissonance is involved in the poor awareness of schizophrenia smokers of the harmful consequences of tobacco smoking. Larger studies are needed to replicate the current exploratory finding, while paying greater attention to the potential confounding effect of treatment resistance on the results.

## Ethics Statement

This study was carried out in accordance with the recommendations of the *Regroupement Neuroimagerie Québec* with written inform consent from all subjects. All subjects gave written consent in accordance with the Declaration of Helsinki. The protocol was approved by the *Regroupement Neuroimagerie Québec*.

## Author Notes

SP is holder of the Eli Lilly Canada Chair on schizophrenia research and is a supported member from the Fondation de l’Institut Universitaire en Santé Mentale de Montréal.

## Author Contributions

The manuscript was authored by SP, AT, OLungu, ES, VZ, PL, OLipp, and AM. All authors have had full access to data in the study, have personally reviewed the manuscript, and gave final approval of the version attached.

## Conflict of Interest Statement

Within the last 3 years, SP has received funding from Eli Lilly, Bristo-Myers Squibb, Otsuka, and INSYS Pharmaceuticals; OLipp has received funding from Lunbeck; and ES has received funding from Janssen-Ortho and Hoffmam-La Roche. OLungu, PL, VZ, AT, and AM report no biomedical interests.
