# Supplementary material for: Impaired Coupling between the Dorsomedial Prefrontal Cortex and the Amygdala in Schizophrenia Smokers Viewing Anti-smoking Images
Source: Front Psychiatry. 2017 Jun 19;8:109. doi: 10.3389/fpsyt.2017.00109 (PMC5474956; doi:10.3389/fpsyt.2017.00109)
Supplement: Supplementary file 1 [file table_1.pdf]

Table S1: Brain activations in schizophrenia patients and healthy controls

Brain region	R/L	BA	Talairach coordinates			Max t-value[1]	Volume (mm^3)
			x	y	z		
			Tobacco > Neutral				
Dorsomedial prefrontal gyrus	L	9	-3	56	43	5.4	1184
Amygdala	L	–	-21	-7	-8	6.2	2793
Inferior frontal gyrus	L	47	-48	29	-2	4.9	1241
Middle frontal gyrus	L	46	-51	32	22	6.1	2977
Inferior Occipital Gyrus/ Fusiform gyrus	R	18/20	24	-88	-5	10.4	51878
Cerebellum/ Uvula	R	–	6	-67	-29	5.8	3062
Inferior temporal gyrus/ Middle occipital gyrus/ Fusiform gyrus	L	37/20	-42	-67	-5	11.8	58554

Abbreviations: R/L = right/left; BA= Brodmann area; [1] False Discovery Rate corrected at p<0.005 brain-wise.
