# Supplementary material for: Impaired Coupling between the Dorsomedial Prefrontal Cortex and the Amygdala in Schizophrenia Smokers Viewing Anti-smoking Images
Source: Front Psychiatry. 2017 Jun 19;8:109. doi: 10.3389/fpsyt.2017.00109 (PMC5474956; doi:10.3389/fpsyt.2017.00109)
Supplement: Supplementary file 2 [file image_1.pdf]

Figure S1. Influence of stimuli content on BOLD signal.

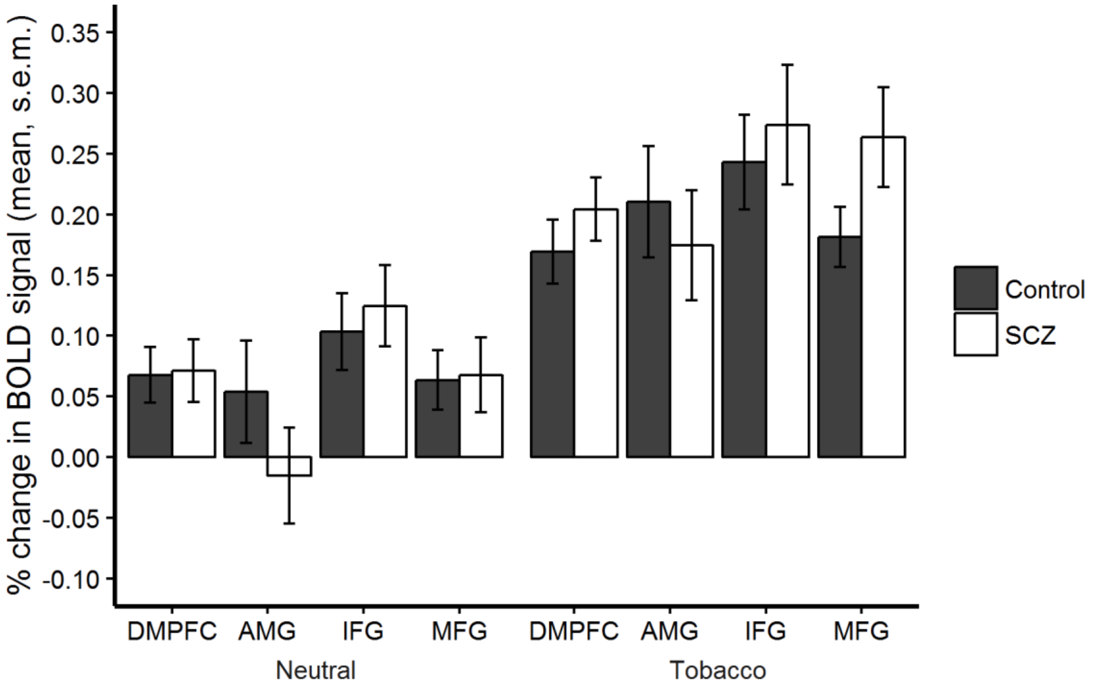

Abbreviations: AMG, amygdala; DMPFC, dorsomedial prefrontal cortex; IFG, inferior frontal gyrus; MFG, middle frontal gyrus; SCZ, schizophrenia patients; Control = healthy control participants.
